# Inositol hexakisphosphate increases the size of platelet aggregates

**DOI:** 10.1016/j.bcp.2018.12.011

**Published:** 2019-03

**Authors:** Maria A. Brehm, Ulrike Klemm, Christoph Rehbach, Nina Erdmann, Katra Kolšek, Hongying Lin, Camilo Aponte-Santamaría, Frauke Gräter, Bernhard H. Rauch, Andrew M. Riley, Georg W. Mayr, Barry V.L. Potter, Sabine Windhorst

**Affiliations:** a Department of Pediatric Hematology and Oncology, University Medical Center Hamburg-Eppendorf, Hamburg, Germany; b Department of Biochemistry and Signal Transduction, University Medical Center Hamburg-Eppendorf, Martinistrasse 52, D-20246 Hamburg, Germany; c Molecular Biomechanics Group, Heidelberg Institute for Theoretical Studies, Heidelberg, Germany; d Institute of Pharmacology, University Medicine Greifswald, Ernst-Moritz-Arndt University, Felix-Hausdorff-Str. 3, 17487 Greifswald, Germany; e Medicinal Chemistry & Drug Discovery, Department of Pharmacology, University of Oxford, Mansfield Road, Oxford OX1 3QT, United Kingdom

**Keywords:** Platelets, Inositol hexakisphosphate, Fibrinogen, Aggregation

## Abstract

The inositol phosphates, InsP_5_ and InsP_6_, have recently been identified as binding partners of fibrinogen, which is critically involved in hemostasis by crosslinking activated platelets at sites of vascular injury. Here, we investigated the putative physiological role of this interaction and found that platelets increase their InsP_6_ concentration upon stimulation with the PLC-activating agonists thrombin, collagen I and ADP and present a fraction of it at the outer plasma membrane.

Cone and plate analysis in whole blood revealed that InsP_6_ specifically increases platelet aggregate size. This effect is fibrinogen-dependent, since it is inhibited by an antibody that blocks fibrinogen binding to platelets. Furthermore, InsP_6_ has only an effect on aggregate size of washed platelets when fibrinogen is present, while it has no influence in presence of von Willebrand factor or collagen. By employing blind docking studies we predicted the binding site for InsP_6_ at the bundle between the γ and β helical subunit of fibrinogen.

Since InsP_6_ is unable to directly activate platelets and it did not exhibit an effect on thrombin formation or fibrin structure, our data indicate that InsP_6_ might be a hemostatic agent that is produced by platelets upon stimulation with PLC-activating agonists to promote platelet aggregation by supporting crosslinking of fibrinogen and activated platelets.

## Graphical abstract

**Figure:**
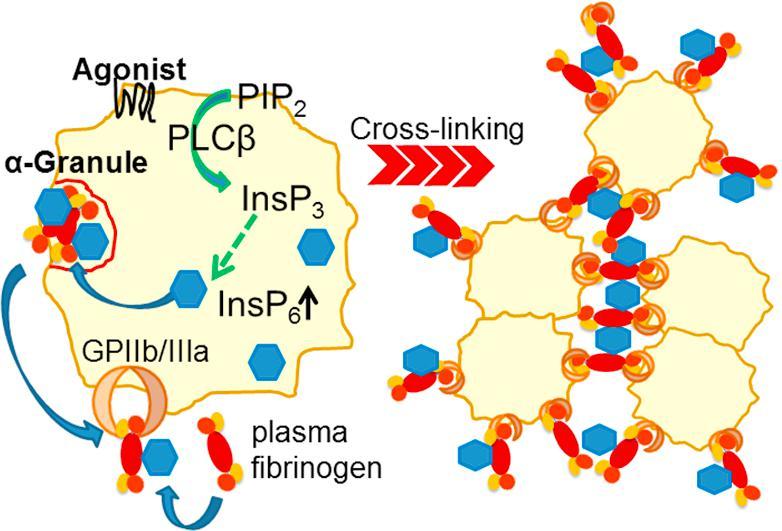

## 1 Introduction

**Fig. 1:**
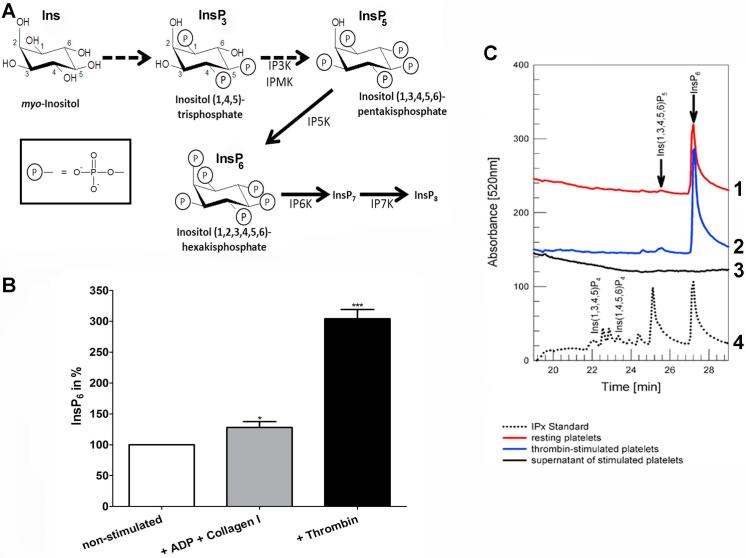
Synthesis of InsP_6_ in mammalian cells. (A) Inositol phosphates are derived from the cyclitol inositol (Ins). In mammalian cells the synthesis of soluble InsPs starts with inositol 1,4,5-trisphosphate (InsP_3_) which is released from phosphatidylinositol 4,5-bisphosphate at the plasma membrane upon PLC activation. InsP_3_ is phosphorylated to inositol 1,3,4,5,6-pentakisphosphate (InsP_5_) by inositol 1,4,5-trisphosphate 3-kinase (IP3K) and inositol multikinase (IPMK), then inositol 1,3,4,5,6-pentakisphosphate 2-kinase (IP5K) performs the last phosphorylation step to generate inositol 1,2,3,4,5,6-hexakisphosphate (InsP_6_), which can be phosphorylated to pyrophosphates InsP_7_ and InsP_8_. The negative charge increases with increasing phosphorylation (℗ symbolizes the phosphate group (insert)). (B and C) Inositol phosphates were extracted by trichloroacetic acid from lysates of washed resting (white) and washed platelets stimulated either with ADP (10 µM) and collagen I (50 µg/ml) in presence of fibrinogen (300 µg/ml) (grey) or stimulated only with 2U/ml thrombin (black) and analyzed by metal dye detection (MDD)-HPLC. Mean values + SEM of three independent experiments are shown. *P*-values ^*^<0.05, ^***^<0.0001. (C) MDD-HPLC chromatograms of one representative experiment out of three independent experiments are shown staggered in panel C. Chromatrogram 1: Analysis of InsPs in non-stimulated platelets, 2: Analysis of InsPs in thrombin-stimulated platelets, 3: Analysis of InsPs in the supernatant of stimulated platelets, 4: InsP standard for comparison of retention times. (For interpretation of the references to color in this figure legend, the reader is referred to the web version of this article.)

Inositol phosphates (InsPs) are derived from the cyclitol inositol, which can be phosphorylated at one or more of its six hydroxyl groups [1] (Fig. 1A). In mammalian cells the synthesis of soluble InsPs starts with inositol 1,4,5-trisphosphate (InsP_3_), which is released from phosphatidylinositol 4,5-bisphosphate (PIP_2_) at the plasma membrane upon PLC activation. InsP_3_ can be phosphorylated to inositol 1,3,4,5,6-pentakisphosphate (InsP_5_) by inositol 1,4,5-trisphosphate 3-kinase (IP3K) and inositol phosphate multikinase (IPMK), then inositol 1,3,4,5,6-pentakisphosphate 2-kinase (IP5K) performs the last phosphorylation step to generate inositol 1,2,3,4,5,6-hexakisphosphate (InsP_6_), which can be pyrophosphorylated to InsP_7_ and InsP_8_ (Fig. 1A) [2].

Inositol phosphates are present in all tissues and can serve as second messengers with a broad spectrum of functions. For example, InsP_3_ releases Ca^2+^ from intracellular stores [3]. InsP_5_ mainly serves as a precursor for the production of InsP_6_. With intracellular concentrations of up to 100 µM [4], InsP_6_ is the most abundant and versatile inositol phosphate within mammalian cells. Intracellular functions include serving as a cofactor for DNA-dependent protein kinase activity in non-homologous end-joining [5] and as an essential folding factor for mRNA and tRNA editing enzymes [6]. InsP_5_ and InsP_6_ further participate in chromatin remodeling [7], [8], [9].

Recently, InsP_5_ and InsP_6_ have been described by some of us to specifically bind to fibrinogen [10]. One of the main functions of this protein lies in hemostasis by crosslinking platelets at the site of vascular injury. Activated platelets bind plasma fibrinogen and they release platelet-fibrinogen that is rebound to their surface [11]. Furthermore, fibrinogen acts as a precursor of fibrin, the major component of blood clots. Here, we investigated the putative role of InsPs in fibrinogen-dependent platelet aggregation.

## 2 Materials and methods

### 2.1 Chemicals

InsP_5_ (triethylammonium salt) was synthesised as previously reported [12]. InsP_3_ (potassium salt) was purchased from Buchem B.V. (Apeldoorn, The Netherlands)_,_ InsP_6_ (potassium salt) from Millipore (Darmstadt, Germany), apyrase from potato and human fibrinogen (purified from plasma) were purchased from Sigma Aldrich (Munich, Germany), fibrinogen from human plasma Alexa Fluor 488 conjugate from Thermo Fisher Scientific (Darmstadt, Germany), human α-thrombin from Haematologic Technologies Inc (Essex Junction, VT, USA), t-PA from Haemachrom (Essen, Germany), Glu-Plasminogen from Enzyme Research Laboratories (South Bend, IN, USA), human collagen type III from Southern Biotech (Birmingham, AL, USA) and May-Grünwald solution from Carl Roth (Karlsruhe, Germany). Chemicals for all buffers described below were purchased from Sigma Aldrich (Munich, Germany).

### 2.2 Blood sampling

The study was conducted in conformity to the Declaration of Helsinki [13] and to The International Conference on Harmonisation of Technical Requirements for Registration of Pharmaceuticals for Human Use (ICH) Guidelines, available at http://www.ich.org, accessed in October 2010. The study was approved by the local ethics committee of the University Medicine Greifswald. Appropriate informed consent was obtained from all subjects. The donors had not taken medication known to affect platelet function for at least ten days before blood sampling. The first 2 ml of blood were discarded.

### 2.3 Preparation of washed blood or platelets

To prepare washed blood, whole blood was collected using sodium citrated blood vacuum syringes (Sarstedt, Nümbrecht, Germany) and apyrase was added to a final concentration of 1.3 U/ml. To prepare large amounts of washed platelets, buffy coats (kindly provided by the Transfusion Medicine, UKE) were diluted 1:1 with modified calcium-free Tyrode’s buffer (137 mM NaCl, 2.7 mM KCl, 0.48 mM NaH_2_PO_4_, 2.7 mM glucose, 5 mM Hepes, pH 6.5) and apyrase was added to a final concentration of 0.65 U/ml. All samples were centrifuged at 1590 × g for 15 min at room temperature (RT) with reduced deceleration. After aspiration of the supernatant, the platelet-poor plasma was replaced with modified calcium-free Tyrode’s buffer to its initial volume. In each following wash step the concentration of apyrase added was reduced by 50%, the last wash contained no apyrase. Centrifugation was performed as above. After four wash steps the blood samples were adjusted to the initial volume with modified calcium free Tyrode’s buffer pH 7.4 containing 5% bovine serum albumin (BSA). Buffy coats were adjusted to ¾ of the initial volume with modified calcium free Tyrode’s buffer containing 1% bovine serum albumin, pH 7.4, after the third wash, centrifuged for 10 min at 490 × g and the platelet-rich buffer fraction was separated from the hematocrit. For activation the platelets were re-calcified with CaCl_2_ (final conc. 1 mM) and stimulated either with human α-thrombin (2 U/ml) or ADP (10 µM) and collagen I (50 µg/ml) in presence of fibrinogen (300 µg/ml).

### 2.4 Inositol phosphate analysis by metal dye detection (MDD)-HPLC

Platelets were washed and stimulated with human α-thrombin or with ADP and collagen as described above. Inositol phosphates were extracted by trichloroacetic acid as previously described [14] and analyzed by MDD-HPLC [15].

### 2.5 Cell fractionation

On average 2*10^10^ washed platelets (see above) were diluted in 500 µl buffer (10 mM Tris-HCl pH 7.0 + 250 mM sucrose) and sonicated 5 × 5 s at 15% intensity (Bandelin Sonoplus GM70 sonicator). After addition of 1 ml buffer, the sample was stepwise centrifuged as previously described [16].

### 2.6 Enrichment of dense and α-granules

To enrich dense granules, platelets were isolated from buffy coats and washed as described above. A 15 ml suspension of 300 × 10^3^ platelets/µl was fractionated by Histodenz gradient as described by Hernandez-Riuz et al. (2007) [17]. The pellet fraction was extracted by TCA and InsPs were analyzed by MDD-HPLC [14]. In order to separate α-granules, platelets were fractioned by differential centrifugation *in a* sucrose gradient as previously described [16]. The pellets obtained after each centrifugation step were extracted by TCA and InsPs were analyzed by MDD-HPLC [14]. See also 2.5.

### 2.7 Analysis of InsP_6_ associated with the outer platelet plasma membrane

Washed platelets (40 ml with 8*10^5^ platelets/µl) were stimulated with 2 U/ml human α-thrombin and 1 mM CaCl_2_, incubated for 30 min at 37 °C and collected by centrifugation at 1590 × g for 3 min at RT. The cells were resuspended in 500 µl wash buffer (Ca-free Tyrode’s buffer (±10 mM EDTA)), shaken for 15 min at 37 °C and 500 rpm and centrifuged (1590 × g, 15 min, RT). The supernatant was kept on ice. The cell suspension was washed thrice more with 100 µl wash buffer. Then 10% trichloroacetic acid (TCA) was added to the combined supernatants and the samples were prepared for and analyzed by MDD-HPLC as previously described [14], [15].

### 2.8 Cone and Plate (let) analysis (CPA)

To perform cone and plate(let) analysis (CPA) we employed the Impact-R device [18], [19] (DiaMed, Switzerland). Citrated whole blood was collected from at least three volunteers and allowed to rest for 45 min at RT. Then, 16 µM InsPs (InsP_3_, InsP_5_ or InsP_6_) or the corresponding volume of PBS in absence or presence of 2.8 µg/ml abciximab (ReoPro®, Lilly Deutschland GmbH, Bad Homburg, Germany) were added and mixed for 1 min at 10 rpm on the tube rotator of the Impact-R device.

When using washed blood, fibrinogen (1.5 mg/ml) was added to facilitate aggregate adhesion. CPA samples contained 16 µM InsPs (InsP_3_, InsP_5_ or InsP_6_) with or without either additional 1.5 mg/ml fibrinogen, 10 µg/ml rVWF (produced as previously described [20]), or 3 µg/ml human collagen type III (Southern Biotech, Biozol, Eching, Germany). After an incubation of 5 min, the samples were mixed at 10 rpm for 1 min.

For CPA, 130 µl of each sample were subjected to flow for 2 min at a defined shear rate of 720 rpm (corresponding to 1800 s^−1^). Subsequently, the wells were washed with PBS, stained with May-Grünwald solution (Roth, Karlsruhe, Germany) for 1 min and further analyzed with the internal image analyzer of the Impact-R device. Platelet aggregation was evaluated by examining the average size (AS) of surface-bound objects [21].

### 2.9 Docking simulations

The coordinates of fibrinogen were taken from its crystal structure (PDB ID. 3GHG, [22]). The fibrinogen dimer (chains A-F), with the monomers arranged tail-to-tail, were considered. Each monomer consisted of three subunits (α, β, and γ). Covalently attached sugars and cations were removed from the dimer. N-termini were capped by acetyl moieties in PyMol [23] to remove artificial positive charges due to truncation of the amino-acid chains. The 3D structure of InsP_6_ was also taken from the Protein Data Bank (PDB ID. 5ICN, [24]). Both the fibrinogen and the InsP_6_ structures were prepared for docking using the AutoDockTools (ADT) software package [25]. Polar hydrogen atoms were added to fibrinogen because only polar hydrogens are considered by AutoDock. Gasteiger charges were assigned to all atoms. A grid box of 650 × 200 × 300 Ångstrom^3^ was considered with a grid spacing of 0.375 Ångstrom in all dimensions. It was centered at the point (x,y,z) = (−45, 2, −20) (in Ångstrom units) using the Cartesian coordinates from the original pdb files. The box covered one whole monomer (α, β, and γ) and about one fifth of a second monomer. This ensured that the dimer interface entered into the calculation. Blind molecular docking calculations were carried out with AutoDock 4 (version 4.2.6) [26] utilizing a Lamarckian genetic algorithm. All the parameters were kept as default except the number of genetic algorithm runs which was set to 50.

### 2.10 Fibrin polymerization

Fibrin polymerization was investigated in plasma. Platelet-poor plasma (PPP) was prepared from citrated whole blood by centrifugation (1590 × g, 15 min, RT) and the following reaction mix was prepared: 30 µl PPP, 3.8 µl InsPs (16 µM) 1.2 µl CaCl_2_ (20 mM), and 12 µl fibrinogen conjugated with Alexa Fluor 488 (125 µg/ml). All given concentrations are final concentrations, the final volume was adjusted to 60 µl with TBS. Polymerization was started by addition of human α-thrombin (final concentration: 0.175 U/ml). (Protocol kindly provided by Robert Ariëns and Fraser Macrae, University of Leeds, UK).

After addition of human α-thrombin the samples were immediately transferred into a channel of an Ibidi µ-slide VI^0.4^ (Ibidi, Martinsried, Germany). After fibrin network formation was completed, Z-stacks with 20 slices of 1 µm were recorded at RT with a confocal microscope (TCS SP5, Leica, Wetzlar, Germany) using an HC PL APO CS2 63.0 × 1.40 OIL UV objective, a 3× digital zoom and the following settings: image size of 512 × 512, laser power of the 488 laser set to 5%. 3D reconstruction of the Z-stacks was performed using ImageJ [27]. Three independent experiments with plasma of three donors were performed.

### 2.11 Turbidity analysis of fibrin polymerization

Polymerization of fibrin was studied by turbidity analysis as described [28]. In brief, fibrinogen (0.5 mg/ml), CaCl_2_ (5 mM) and InsPs (16 µM) were diluted in TBS and premixed in 96-well plates in triplicate. Thrombin (0.1 U/ml) was added to initiate clotting, and absorbency was measured at 340 nm, every 12 s (s) for 2 h at room temperature, using a Tecan infinite M200 microtiter plate reader (Tecan Group Ltd., Männedorf, Switzerland). All given concentrations are final concentrations. Three independent experiments were each performed in triplicate.

### 2.12 Fibrinolysis analysis by turbidity

Fibrin clot lysis was studied using a turbidity assay as described by Duval et al. [29]. In brief, fibrinogen (0.5 mg/ml), CaCl_2_ (5 mM), tissue plasminogen activator (t-PA) (100 pM), Glu-plasminogen (0.24 µM) and InsPs (16 µM) were diluted in TBS, mixed and added to 96-well plates in triplicate. Human α-thrombin (0.1 U/ml) was used to initiate clotting, and changes in absorbency were monitored at 340 nm, every 12 sec for 2 h at 24 °C, using the Tecan infinite M200 microtiter plate reader. All given concentrations are final concentrations. Three independent experiments were each performed in triplicate. Lysis rates were calculated by determining the slope of the polymerization curve at the point of its steepest inclination. Lysis rate were expressed as change in optical density per min (ΔOD/min).

### 2.13 Measurement of thrombin formation

Calibrated automated thrombin generation (CAT) was determined by the method described by Hemker et al. [30] Citrated blood was obtained from healthy donors and PRP was prepared by centrifugation at 1000 × g for 45 s at RT. 40 µl PRP were incubated in round-bottom 96-well microtiter plates (Falcon®, VWR, Darmstadt, Germany) with InsPs (16 µM InsP_3_, InsP_5_, InsP_6_) and tissue factor (3 pM) at 37 °C. ADP (10 µM) served as positive control. Thrombin generation was started by automated addition of 20 μl of 20 mM Hepes (pH 7.35), containing 60 g/l BSA (Sigma), 100 mM CaCl_2_ and 2.5 mM of the thrombin-specific fluorogenic substrate (Z-Gly-Gly-Arg-AMC; Bachem, Weil am Rhein, Germany). Fluorescence intensity was detected at wavelengths of 390 nm (excitation filter) and 460 nm (emission filter) using a Fluoroscan Ascent® reader (Thermo Fisher Scientific, Braunschweig, Germany). Thrombin generation was determined in comparison with the thrombin calibrator (Stago). Measurement curves were calculated using the Thrombinoscope® Software (Stago Deutschland GmbH, Düsseldorf, Germany) and displayed thrombin activity over time. All experiments were carried out in quadruplicate at 37 °C, measurements usually lasted 90 min. The relevant parameters that can be derived from CAT are lag time, endogenous thrombin potential (ETP) corresponding to the area under the CAT curve, peak height of thrombin corresponding to the maximal amount of thrombin that can be generated by the plasma sample during the thrombin burst [30].

### 2.14 Light transmission aggregometry

Light Transmission Aggregometry (LTA) was performed using a Chronolog-700 aggregometer (Chrono-log Corporation, Havertown, PA, USA). Platelet-rich plasma (PRP) was generated from citrated whole blood by centrifugation in a VWR Mega Star 600R centrifuge (VWR, Darmstadt, Germany), rotor 75,005,701 (VWR), at 300 rpm and RT for 10 min. PRP was centrifuged at 3000 rpm for 5 min at RT to generate platelet-poor plasma (PPP). PPP was used as blank. Final concentrations of 16 µM InsP_3_, 16 µM InsP_5_ and 16, 160 and 1600 µM InsP_6_, were added to PRP and turbidity was recorded for 10 min. Afterwards, baseline was reset, ADP was added to a final concentration of 10 µM and turbidity was again recorded for 10 min. Three independent experiments were performed.

### 2.15 Statistics

Unpaired *t*-tests were performed using GraphPad Prism version 5.02 for Windows (GraphPad Software, San Diego CA, USA). ^*^p < 0.05; ^**^p < 0.005; ^***^p < 0.0005. Always mean values + SEM of at least three independent experiments are shown.

## 3 Results

### 3.1 Thrombin stimulation increases the InsP_6_ concentration in platelets

We strived to determine the physiological role of the previously described InsP-fibrinogen interaction [10]. Since highly phosphorylated InsPs carry multiple negative charges (Fig. 1A), they might promote fibrinogen-fibrinogen and/or fibrinogen-platelet crosslinking in response to platelet activation. Such a mechanism would require InsP generation in platelets upon stimulation. Using metal dye detection (MDD)-HPLC we analyzed InsPs isolated from resting and stimulated platelets and present here the first direct proof of InsP_6_ synthesis in platelets. Intriguingly, the InsP_6_ content was increased by 204% ± 15% upon thrombin-mediated activation (Fig. 1B, an exemplary MDD-HPLC chromatogram of one representative experiment out of three independent experiments are shown staggered in Fig. 1C). Assuming the volume of a single platelet to be about 10 fl, the overall InsP_6_ concentration in the platelet pellet after stimulation was calculated from the number of used platelets and the area under the InsP_6_-peak to be 16 µM. As expected the concentration of the InsP_6_ precursor, InsP_5_, was adequately increased by about 2.3-fold (Fig. 1C). Since ADP and collagen I were shown to also activate the inositol phosphate generating PLC signalling pathway [31], [32], we further tested the response of washed platelets to 10 µM ADP and 50 µg/ml collagen I. For this milder stimulation the presence of fibrinogen is necessary for aggregation [33], we thus added 300 µg/ml of this plasma protein to the experiments and observed a mild increase of InsP_6_ by 28% ± 9% (Fig. 1B). These data point towards a role of InsP_6_ in platelet function after their activation.

### 3.2 InsP_6_ associates with the plasma membrane of stimulated platelets

**Fig. 2:**
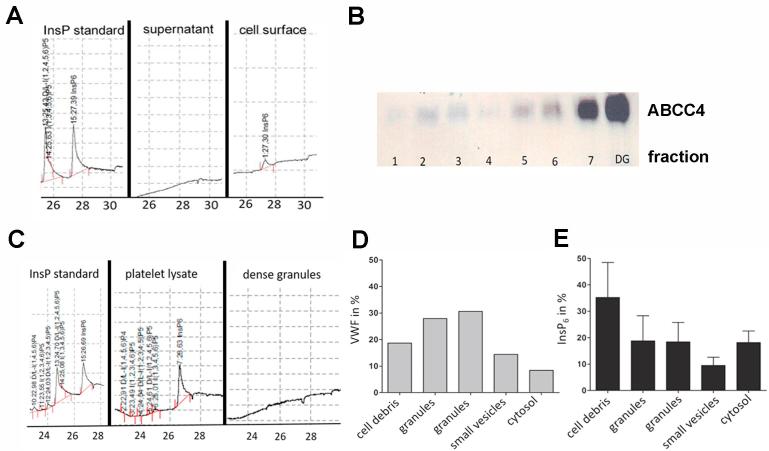
Storage and secretion of InsP_6_ by platelets. (A) Thrombin-stimulated platelets were washed to detach surface bound InsP_6_. The platelet supernatant and the wash was analyzed by MDD-HPLC. InsP_6_ (standard shown left) was detected only in the wash fraction (cell surface, right). (B) Platelets were fractioned by histodenz gradient and enrichment of dense granules in the pellet (DG) was analyzed by Western blotting using an antibody against the dense granule marker ABCC4. (C) InsPs were extracted from from whole platelets (middle) as well as the dense granule containing pellet (right) with TCA and analyzed by MDD-HPLC. InsP standard is shown left. (D and E) Platelets were fractioned by a sucrose gradient and after differential centrifugation the pellets were analyzed for the α-granule containing fractions by VWF ELISA (D) and InsPs were analyzed by MDD-HPLC (E).

A putative role of InsP_6_ in fibrinogen-fibrinogen and/or fibrinogen-platelet crosslinking would require InsP_6_ secretion. Since we did not detect InsPs in the supernatant of stimulated platelets (Figs. 1C and 2A), we hypothesized that they might be bound to the platelet plasma membrane. To analyze this hypothesis, stimulated platelets were washed to remove cell surface-bound InsPs. MDD-HPLC analysis of the wash supernatants revealed a clear InsP_6_ signal (Fig. 2A). No other InsPs were detected. These data suggest that InsP_6_ is secreted by stimulated platelets and is subsequently bound to their surface.

In order to determine the mechanism of InsP_6_ secretion by platelets, we examined if InsP_6_ may be present in dense or α-granules. These granules serve as stores for platelet-derived factors involved in hemostasis, which are secreted after platelet stimulation (reviewed in [34]). First, dense granules were isolated by histodenz density gradient centrifugation by which the dense granule-rich fraction remains in the pellet [17]. Enrichment of dense granules was validated by Western blotting employing an antibody against the dense granule marker nucleotide transporter MRP4 (ABCC4) [35] (Fig. 2B) and InsPs were extracted and analyzed by MDD-HPLC. As positive control InsPs were also analyzed from whole platelet lysate. However, no InsPs could be detected in the dense granule fraction (Fig. 2C). Next, we wanted to enrich α-granules but for this approach matrices like metrizamide are used (reviewed in [36]) which cannot be loaded to HPLC columns. As an alternative approach, a sucrose density gradient was used to collect different cellular compartments after differential centrifugation in the pellet [16]. Enrichment of α-granules was analyzed by ELISA for von Willebrand factor (VWF), which is stored in α-granules [37], [38]. The highest VWF signals were found in fraction 2 and 3 (Fig. 2D). Therefore, these fractions were termed granule fractions. Analysis of InsP_6_ after differential centrifugation revealed that the cell debris (non-lysed cells and debris) contained the highest amount of InsP_6_ (about 35%), while 37% of InsP_6_ were found in granule fractions and 18% inside the cytosol (Fig. 2E). Thus, it seems that a fraction of InsP_6_ is synthesized inside the cytosol and subsequently transported into α-granules.

In conclusion our data reveal that platelets secrete InsP_6_, most likely by α-granule-mediated release.

### 3.3 InsP_6_ influences platelet aggregate size in a fibrinogen-dependent manner

**Fig. 3:**
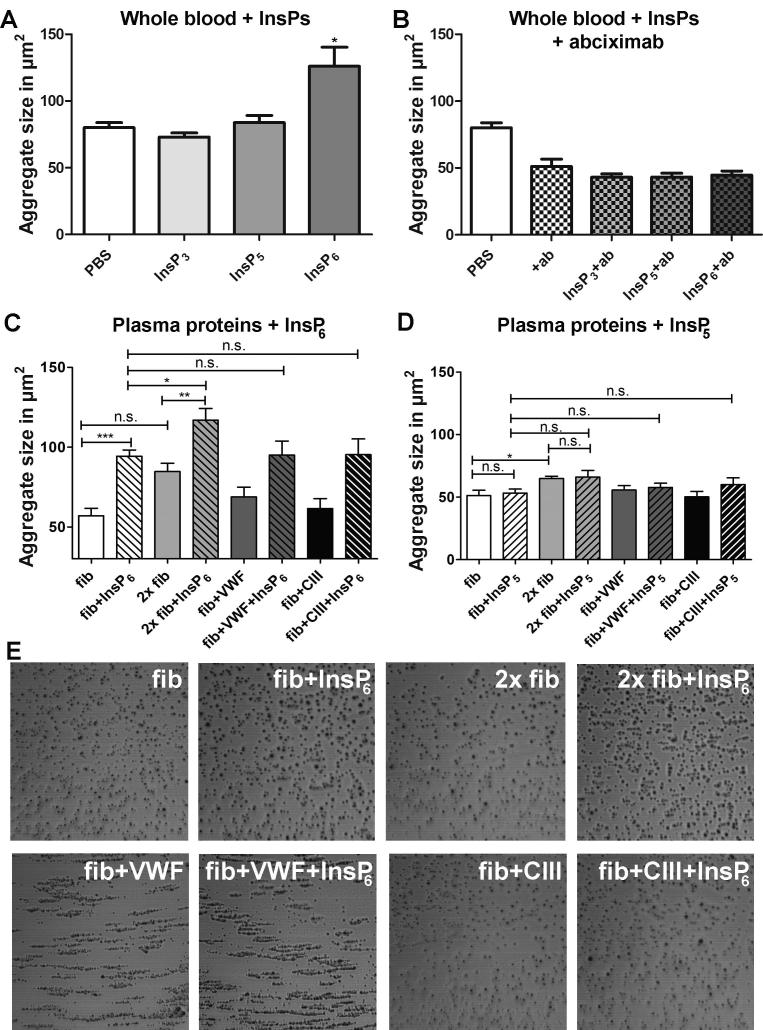
InsP_6_ increases platelet aggregate size in a fibrinogen-dependent manner. (A, B) Whole blood was incubated with 16 µM InsP_3_ (light grey), InsP_5_ (grey), InsP_6_ (dark grey) or PBS (white) in absence (A) or presence (B) of 2.8 µg/ml of the GPIIb/IIIa blocking antibody abciximab for 1 min while rotating at 10 rpm. Then the samples were subjected to flow for 2 min at 720 rpm (=1800 s^−1^) in the cone and plate analyzer Impact-R. After washing and staining with May-Grünwald solution the average size of surface-bound platelet aggregates was analyzed using the internal image analyzer. (C and D) Washed blood supplemented either only with 1.5 mg/ml fibrinogen (white) or with additional 1.5 mg/ml fibrinogen (light grey), 10 µg/ml VWF (dark grey), 3 µg/ml human collagen type III (CIII, black) ±16 µM InsP_6_ (panel C, striped columns with InsP_6_) or ±16 µM InsP_5_ (panel D, striped columns with InsP_5_) was incubated for 5 min and then mixed for 1 min while rotating at 10 rpm. Then the samples were subjected to flow for 2 min at 720 rpm (=1800 s^−1^) in the cone and plate analyzer Impact-R. After washing and staining with May-Grünwald solution the average size of surface-bound platelet aggregates was analyzed using the internal image analyzer. Mean values ± SEM of three independent experiments (each performed in duplicates) are shown. Unpaired *t*-test: ^*^p < 0.05, ^**^p < 0.005 ^***^p < 0.0001; n.s.: not statistically significant. (E) One representative image of each series of the experiments summarized in panel C are shown. (For interpretation of the references to color in this figure legend, the reader is referred to the web version of this article.)

We next investigated if InsP_6_ has an effect on fibrinogen-dependent platelet aggregation. Thus, whole blood was incubated with 16 µM of InsP_6_ for 1 min. As controls PBS, InsP_3_ and InsP_5_ were employed. Aggregate size was measured by the cone and plate analyzer, Impact-R. Compared to the PBS control, InsP_3_ and InsP_5_ did not alter the aggregate size but we found a significant increase in response to InsP_6_ by 57% ± 27% (Fig. 3A). To analyze if this effect is also mediated by binding of InsP_6_ to fibrinogen, we added the GPIIb/IIIa (integrin α_IIb_β_3_) blocking antibody abciximab. Since fibrinogen binding is crucial for platelet aggregate formation, this procedure decreased aggregate size also in the control (Fig. 3B). The fact that under these conditions presence of InsP_6_ did not lead to bigger aggregates than in the controls, confirms the fibrinogen dependence of the InsP_6_-mediated effect.

To further substantiate this finding, we depleted whole blood of all serum proteins by several wash steps and determined aggregate size in the presence of InsP_6_ (Fig. 3C) after selective addition of the platelet binding proteins fibrinogen, von Willebrand factor (VWF), and collagen type III. Since cone and plate analysis (CPA) requires fibrinogen to facilitate aggregate adhesion, we reconstituted all samples with a low physiological fibrinogen concentration of 1.5 mg/ml (Fig. 3C, white column). Addition of 16 µM InsP_6_ increased aggregate size by 65% (from 57.0 ± 4.7 µm to 94.3 ± 3.9). This effect was enhanced to 105% ± 15% (from 57.0 ± 4.7 µm to 117.0 ± 7.2 µm)) only by addition of more fibrinogen (to a final concentration of 3 mg/ml) while VWF and collagen type III (Fig. 3C) had no additional enhancing effect. These data provide further evidence that InsP_6_ exclusively affects aggregation through interaction with fibrinogen. InsP_5_ again did not change aggregate size (Fig. 3D) confirming that the observed effect is highly specific for InsP_6_.

### 3.4 Putative InsP_6_ binding sites of fibrinogen

**Fig. 4:**
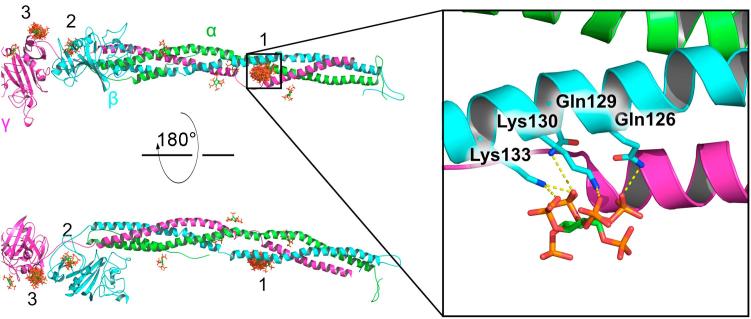
Putative InsP_6_ binding sites at fibrinogen revealed by molecular docking. Fibrinogen is depicted as a cartoon, highlighting the different subunits α, β, and γ by different colors. InsP_6_ is predicted to bind mainly in three regions (labeled as 1–3). 50 resulting binding poses are overlaid, with InsP_6_ in stick representation. The region 1, in between the helices of subunits γ and β, clusters the majority of the binding poses (31/50), including the best scored pose and 9 out of the top 15 scored poses. The zoom displays the conformation adopted by InsP_6_ at this position (for the highest scored pose). Electrostatic interactions are predicted to favor the binding at this site. Potential stabilizing salt bridges and hydrogen bonds interactions between InsP_6_ and the indicated residues of the β subunit are highlighted by dashed lines.

To gain deeper insights into the mechanism of the InsP_6_-fibrinogen interaction, we predicted putative binding sites of InsP_6_ at fibrinogen by performing blind docking calculations. Our calculations revealed three major potential InsP_6_ binding sites (labelled 1–3 in Fig. 4). 34/50 (68%) of the binding poses targeted the helical region of the β chain (1 and 2 in Fig. 4), while 13/50 (26%) pointed towards the γ subunit (3 in Fig. 4). This result is consistent with a previous study by some of us, that indicated that InsP_6_ binding to fibrinogen might occur in the β chain [10]. Interestingly, the majority of binding poses (31/50) were found at one specific site along the β chain (binding site 1, Fig. 4), in between the helical bundle formed by the γ and the β chains, interacting with residues of the latter. This site also contained the best scored pose (Fig. 4, zoom), as well as, 9 out of the top 15 best ranked poses. InsP_6_ is predicted to be stabilized at this position by salt bridges between its phosphate groups and the amine groups of Lys130 and Lys133, together with hydrogen bonds with the amide groups of Gln129 and Gln126 (Fig. 4, zoom). Therefore, these residues are good candidates for future mutational studies aiming at identifying the InsP_6_ binding site. Taken together, our calculations suggest InsP_6_ to bind to one specific site of fibrinogen: The interface between the γ and the β chains at the bundled helical region of fibrinogen.

### 3.5 Fibrin polymerization is unaffected by InsPs

**Fig. 5:**
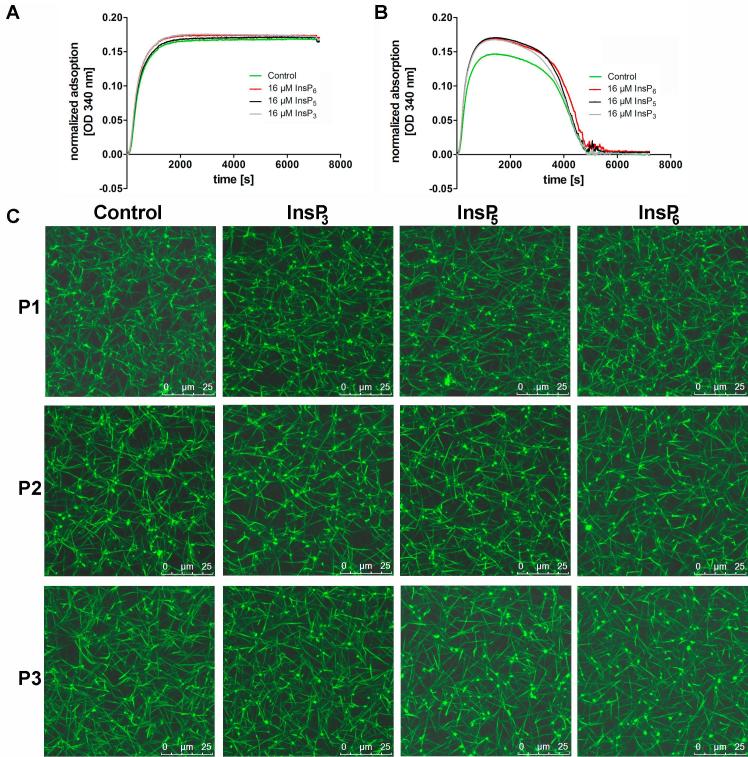
Fibrin polymerization and fibrinolysis in the presence of InsPs. (A) For turbidity analysis of fibrin polymerization, fibrinogen purified from human plasma was incubated with 16 µM InsP_3_ (grey line), InsP_5_ (black line) or InsP_6_ (red line) in the presence of 5 mM CaCl_2_ and 0.1 U/ml human α-thrombin. The control without InsPs is shown by the green line. (B) Fibrinolysis was measured employing an adapted turbidity assay by addition of t-PA (100 pM) and plasminogen (0.24 µM) to the reaction described in (A). Changes in absorbency were monitored at 340 nm, every 12 s for 2 h at room temperature, using a microtiter plate reader. (C) Human plasma from three different donors (P1-P3), CaCl_2_ (5 mM), Alexa Fluor 488 labeled fibrinogen (10%) and 16 µM InsP_3_ (D), InsP_5_ (E) or InsP_6_ (F) were diluted in TBS. After addition of human α-thrombin (0.175 U/ml final concentration), the reaction mixture was immediately transferred into the channel of an Ibidi µ-slide VI^0.4^. After fibrin network formation was completed, Z-stacks with 20 slices of 1 µm were recorded at room temperature using a confocal microscope (TCS SP5, Leica, Wetzlar, Germany) equipped with an HC PL APO CS2 63.0 × 1.40 OIL UV objective (Leica) and the following settings: zoom 3×, image size of 512 × 512, laser power of the 488 lasers was set 5%. 3D reconstruction was performed using the ImageJ software [27]. The scale bars represent 25 µm. (For interpretation of the references to colour in this figure legend, the reader is referred to the web version of this article.)

Since fibrinogen is the precursor of fibrin, we then investigated if thrombin-induced formation of fibrin or its degradation might be influenced by the presence of InsPs. In turbidity measurements with purified fibrinogen neither InsP_3_, InsP_5_ nor InsP_6_ showed an effect on fibrin formation (Fig. 5A). Addition of t-PA and plasminogen to the reaction revealed that InsPs further do not influence the rate of fibrin degradation (Fig. 5B) as no significant difference in the rate of fibrinolysis was detected (ΔOD_340_/min of the control = −0.00591 ± 0.00053; InsP_3_ = −0.00742 ± 0.00219; InsP_5_ = −0.00803 ± 0.00269; (InsP_6_) = −0.00552 ± 0.00134).

Another molecule that is rich in phosphate groups, namely inorganic polyphosphate (polyP), alters fibrin structure. To investigate if InsPs might have a similar effect under more physiological conditions, we induced fibrin polymerization in human plasma samples from 3 different donors by addition of 0.175 U/ml human α-thrombin. Visualization of fibrin was facilitated by incorporation of about 10% fluorescently labeled purified fibrinogen. As shown in Fig. 5C, density of the fibrin network differed between the three donors but none of the added InsPs exhibited an effect on fibrin structure.

### 3.6 InsPs have no direct effect on platelet activation and thrombin generation

Because InsPs might be released by damaged endothelial cells at sites of vascular injury, we further determined if InsPs might also have direct fibrinogen-independent effects on platelets.

**Fig. 6:**
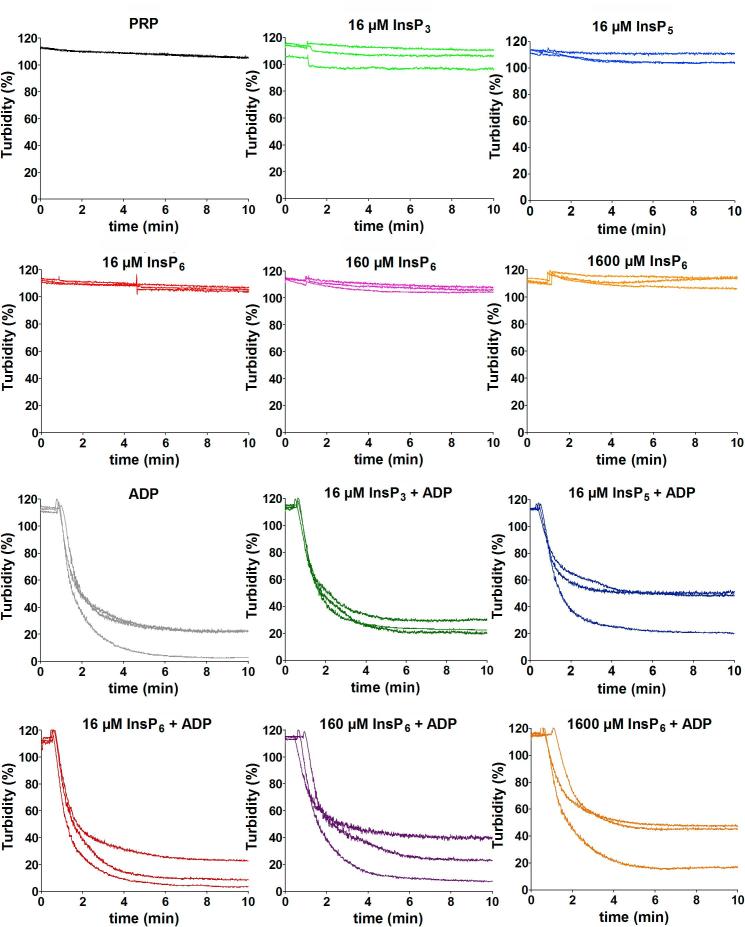
Inoistol phosphates do not activate platelet aggregation. Light Transmission Aggregometry (LTA) was performed using a Chronolog-700 aggregometer. Platelet-rich plasma (PRP) was generated from citrated whole blood by centrifugation at 300 rpm and RT for 10 min. PRP was centrifuged at 3000 rpm for 5 min at RT to generate platelet-poor plasma (PPP). PPP was used as blank. Final concentrations of 16 µM InsP_3_, 16 µM InsP_5_ and 16, 160 and 1600 µM InsP_6_, were added to PRP and turbidity was recorded for 10 min. Afterwards, baseline was reset, ADP was added to a final concentration of 10 µM and turbidity was again recorded for 10 min. Three independent experiments were performed each and all curves are shown in the indicated panels.

Light transmission aggregometry (LTA) was used to investigate if InsPs can act as direct activators of platelet aggregation. Addition of 16 µM InsP_3_, InsP_5_ and InsP_6_ to platelet-rich plasma (PRP) did not lead to aggregation (Fig. 6); neither did higher InsP_6_ concentrations (160 and 1600 µM). 10 min after InsP addition, we further added 10 µM ADP to confirm aggregatability of the platelets and observed normal aggregation which was not influenced by presence of InsPs (Fig. 6).

**Fig. 7:**
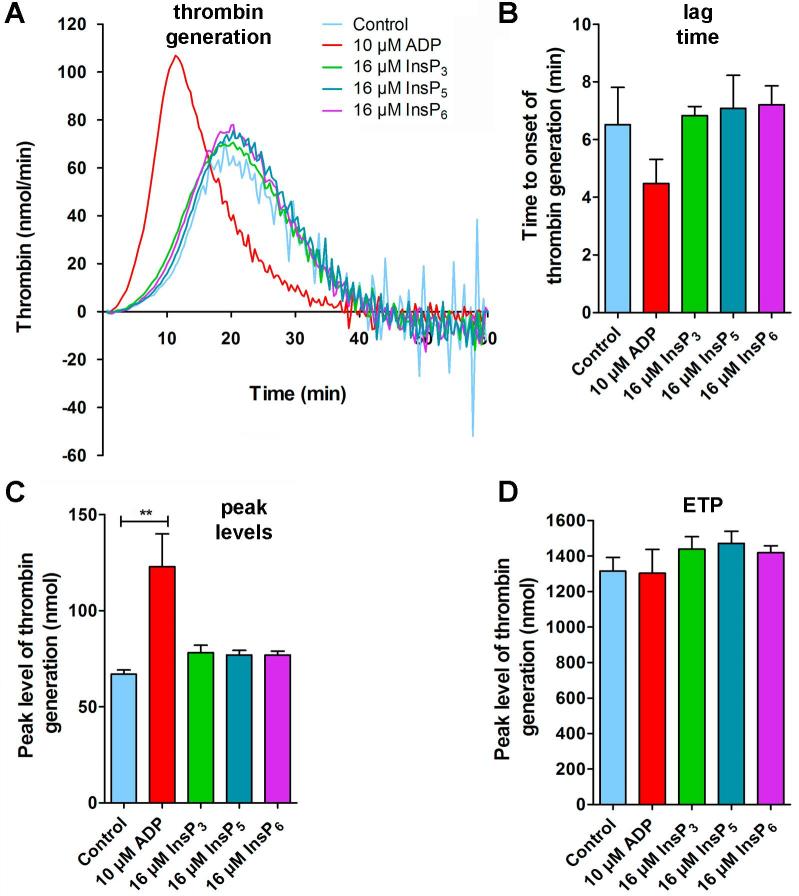
InsPs do not enhance thrombin generation. (A) PRP was incubated with either 16 µM InsP_3_ (green), InsP_5_ (dark blue), InsP_6_ (magenta) or with ADP (10 µM, red) for 10 min. Then, thrombin generation was initiated by addition of CaCl_2_ and tissue factor. Formation of thrombin was determined in quadruplicate by the calibrated automated thrombin generations assay (CAT) as described in the method section. Shown is one representative experiment out of three measurements. (B) The time to onset (lag time) and (C) the peak levels of thrombin generation were quantified by CAT in the indicated groups. (D) The endogenous thrombin potential (ETP, nmol) is a measure for the total thrombin generated within the sample. (B–D) Mean values ± SEM of three independent experiments (performed in quadruplicates) are shown. Statistically significant differences between groups were determined by one-way ANOVA (F (4, 9) = 12, 49; *p* = 0.001). (For interpretation of the references to colour in this figure legend, the reader is referred to the web version of this article.)

To investigate if InsPs affect the binding of coagulation factors to the platelet surface and subsequent generation of thrombin, we measured platelet-induced thrombin formation [39] by analyzing thrombin activity in platelet-rich-plasma (PRP) with and without 16 µM InsPs (InsP_3_, InsP_5_, InsP_6_)_._ Since ADP activates platelets and thus accelerates the onset and enhances peak levels of thrombin formation, addition of ADP served as positive control (Fig. 7A). We did not detect an effect of InsPs on thrombin formation parameters such as lag time (Fig. 7B), peak levels (Fig. 7C) or the overall thrombin generation capacity (ETP) (Fig. 7D). These results indicate that InsPs do not directly activate platelets and have no effect on thrombin generation.

## 4 Discussion

For the first time, we directly demonstrated that platelets produce InsP_6_ and that they increase their InsP_6_ content from about 5 µM to 16 µM upon stimulation with thrombin. The low concentration of InsP_5_ indicates that it serves only as a precursor of InsP_6_, which is then the predominant effector molecule. Stimulation with ADP and collagen I also increased InsP_6_ production suggesting that at least one of these agonists – but most likely both of them as they both activate the inositol-producing PLC pathway [31], [32] – stimulate InsP_6_ production. Together, these data indicate a physiological role for this inositol phosphate during platelet aggregation.

When talking about substances with multiple phosphate groups as players in hemostasis, inorganic polyphosphate (polyP) is the first molecule that comes to mind. Here, we identified another phosphate carrying molecule, namely InsP_6_, with a putative role in hemostasis that is fully independent and different from the function of polyP. PolyP was shown to be a potent modulator of the blood clotting cascade, resulting in accelerated thrombin generation, reversal of the anticoagulant activity of a variety of anticoagulants (e.g. TFPI) and polyP enhances fibrin clot structure and resistance of clots to fibrinolysis (reviewed in [40]). InsP_6_ seems to be only indirectly involved in the function of polyP since it has been shown that knock-down of IP6K, the enzyme that converts InsP_6_ to InsP_7_, leads to decreased levels of polyP in mice [41]. In addition, the routes of synthesis, storage and secretion of InsP_6_ and PolyP are different. In contrast to InsP_6_, which is produced from InsP_3_ after PLC stimulation, polyP is synthesized from ATP. The role of InsP_7_ in the latter process has not yet been uncovered. It has been suggest that InsP_7_ may influence polyP synthesis by regulating ATP uptake into the cell [42] because it has been shown to control the cell’s energy metabolism [43]. Another hypothesis is that InsP_7_ might regulate polyP-kinases, the enzymes that synthesizes polyP [41], [42]. Further, polyP and InsP_6_ are not stored in the same cell compartment. While polyP is stored in dense granules, we did not find InsP_6_ in these substructures. Instead, our data indicate that InsP_6_ is stored in α-granules. Confirmation of these data and elucidation of how InsP_6_ could be transported into these granules will require further investigation in the future. In plants, it has been demonstrated that ABCC type ATP binding cassette (ABC) transporters, also designated MRP5 and MRP4 in *Arabidopsis* and *zea maize*, respectively, are involved in InsP_6_ transport from the cytosol to an extracytosolic compartment [44]. Since also in humans and yeast, members of the ABCC type transporters of unknown function exist [45], we would not rule out such an ATP dependent InsP_6_ transport into platelet granules.

An additional difference between InsP_6_ and polyP is that the latter is released into the circulation after platelet activation and stimulates soluble factors of the coagulation cascade, whereas InsP_6_ remains bound to the platelet surface. In summary, although InsP_6_, as a precursor of InsP_7_, is indirectly involved in the synthesis of polyP, it most likely plays independent roles in hemostasis.

Since InsP_6_ neither exhibits a direct influence on platelet aggregation, thrombin generation nor on the formation, structure or lysis of fibrin, our data indicate that InsP_6_ acts in response to platelet activation and independent of polyP. The first step in primary hemostasis in the arterial circulation is the attachment of VWF to subendothelial collagen that was exposed by the injury. VWF and collagen then recruit platelets. The platelet binding and the presence of agonists, such as thrombin, lead to a variety of signaling events that induce conformational change of the GPIIb/IIIa complex which is then able to bind plasma fibrinogen [46]. In parallel, exocytosis leads to secretion of platelet-stored fibrinogen that rebinds to the platelet plasma membrane [11].

Since our data indicate that InsP_6_ associates with fibrinogen presented at the cell surface and reveal that addition of InsP_6_ leads to an increase of platelet aggregate size, we suggest that InsP_6_ supports and stabilizes the crosslinking between fibrinogen and platelets. This effect is highly specific to InsP_6_, since InsP_3_ and InsP_5_ did not alter platelet aggregate size.

**Fig. 8:**
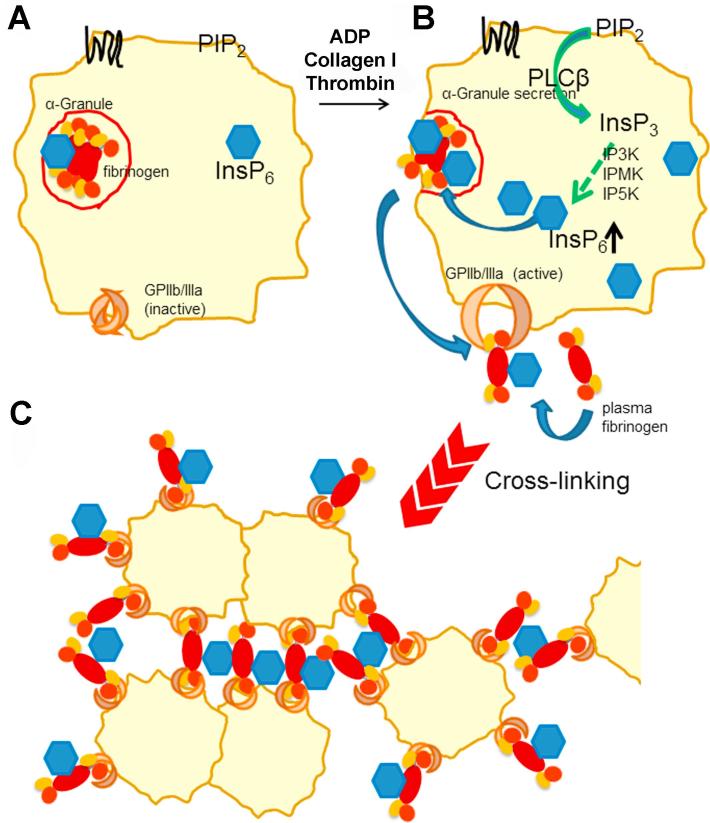
Proposed mechanism of InsP-supported fibrinogen-platelet-crosslinking. (A) InsP_6_ is present in the cytosol and enriched in α-granules. (B) Thrombin, ADP and collagen I stimulation of platelets activates PLC, resulting in generation of InsP_3_ from PIP_2_. InsP_3_ is step-wise phosphorylated to InsP_6_ by inositol phosphate kinases, IP3K, IPMK and IP5K and is at least partially transported into α-granules. Fibrinogen in complex with InsP_6_ is released from platelets and exposed on the plasma membrane where it could recruit plasma fibrinogen. (C) InsP_6_ bound to fibrinogen promotes crosslinking of activated platelets and fibrinogen, thereby stabilizing the growing thrombus.

We thus suggest the following mechanism of InsP_6_ supported fibrinogen-platelet crosslinking (Fig. 8): It is well established that platelet stimulation by thrombin, ADP and collagen I activate PLCβ, resulting in generation of InsP_3_ from PIP_2_ [46]. In mammalian cells, InsP_3_ is generally step-wise phosphorylated to InsP_6_ in the cytoplasm by the inositol phosphate kinases IP3K, IPMK and IP5K [47]. Since thrombin stimulation of platelets activates PLCβ and increases InsP_6_ synthesis, our data indicate that this is also true for platelets (Figs. 1A and 8A). Our cell fractionation data suggest an enrichment of InsP_6_ in α-granules where it presumably binds to fibrinogen. Upon activation the complex is released from platelets and rebound to their surface where it further could recruit plasma fibrinogen. While fibrinogen mainly binds to GPIIb/IIIa on the platelet surface via the ARG sequence in its γ chain [48] and also via the RGD motif in its α chain [49], our blind docking studies indicate that InsP_6_ binds to the β chain of fibrinogen. These binding events could lead to crosslinking of platelet- and plasma-derived fibrinogen, thereby enhancing the interaction between fibrinogen molecules which are bound to platelets (Fig. 8B). This additional interaction could promote and stabilize thrombus formation (Fig. 8C).

InsP_6_ has comprehensively been investigated with regard to its inhibitory effects on growth and invasiveness of a number of cancer types [50]. It has thus been suggested that dietary update of high InsP_6_ concentrations could be beneficial for these individuals [50]. Our data now indicate that increasing the plasma concentration of InsP_6_ by elevated dietary uptake could lead to increased platelet aggregate size. Since patients with cancer have an increased risk of thrombosis [51], it is possible that high InsP_6_ plasma concentrations could further increase this risk. We thus suggest that it might be contraindicative for cancer patients to use InsP_6_ as a dietary supplement.

Our data further suggest the possibility to use new InsP_6_ inhibitors as a novel therapeutic or prevention option for thrombosis. Here, it should be kept in mind that this treatment could also be contraindicative in cancer patients as the blocking of InsP_6_ might increase cancer progression.

In summary, our data reveal that InsP_6_ is a new signaling molecule in platelets and a potential novel player in hemostasis with a physiological role in platelet function by supporting crosslinking of fibrinogen and activated platelets. This is of high relevance because, in future studies, InsP analogues, or similar more drug-like molecules, could be tested on their efficiency to compete with InsP_6_ at its binding sites of fibrinogen without having a crosslinking effect. Such molecules could be of clinical use to destabilize thrombi and thus as novel drugs to prevent thromboembolic events, which occur independent of cancer.
